# BUDDY-UP DYADIC PHYSICAL ACTIVITY (BUDPA) PROGRAM ON HEALTH OUTCOMES OF DEMENTIA CARE DYADS: A FEASIBILITY TRIAL

**DOI:** 10.1093/geroni/igad104.2399

**Published:** 2023-12-21

**Authors:** Doris S F Yu, Polly Li

**Affiliations:** The University of Hong Kong, Hong Kong, Hong Kong; The University of Hong Kong, Hong Kong, Hong Kong

## Abstract

Dementia is a challenging disease experience not only jeopardizes the health of the persons with the condition (PWD) and their family caregivers. In views of the health promoting effects of exercise and its high relevance to promote a meaningful encounter between the dementia care dyads, a pilot feasibility was conducted to examine the effects of a 12-week multi-component Buddy-Up Dyadic Physical Activity (BUDPA) Program. Thirty care dyads were randomized to receive the BUDPA program or wait-list control. Outcome evaluation at baseline, 6 weeks and 12 weeks showed greater improvement in most of the cognitive functional domains (Coden’s d = 0.30-0.87), neuro-psychiatric symptoms (Coden’s d = 0.22), overall mood status (Coden’s d = 0.30) and positive aspects of caregiving (Coden’s d=0.32) among the BUDPA group, though the limited statistical power did not identify significant difference. The qualitative findings from eight care dyads engaged in BUDPA program converged with the outcome evaluation, and BUDPA was described as a ‘physical-based social-cognitive training’. The PwDs were more engaging and attentive in daily life, with less irritability, delusive thoughts and repetitive behaviors. Caregivers reported feelings of vitality and enhanced mood. Converge with the 3-Phase Dyadic Adaptation Model, the care dyads felt more connected and their feeling of ‘being cared for by the PwD is very powerful to reward their effort in caregiving. This finding implies the dyadic exercise is of high fidelity, and a full-scale study is needed to evaluate its effect from a health-promoting, social-interactional and existential paradigms.

